# Supplementary material for: The matrix reasoning item bank (MaRs-IB): novel, open-access abstract reasoning items for adolescents and adults
Source: R Soc Open Sci. 2019 Oct 23;6(10):190232. doi: 10.1098/rsos.190232 (PMC6837216; doi:10.1098/rsos.190232)
Supplement: Supplementary Material [file rsos190232supp1.docx]

**Supplementary Material**

**Supplementary Results**

*Convergent validity (N = 50)*

We here report the results of the first sample of our convergent validity study (N = 50, 36 females, 14 males, mean age = 24.18, *SE* = 0.49, age range 20-35 years) for transparency on multiple rounds of data collection.

To assess convergent validity we inspected the product-moment correlation between MaRs-IB performance (at the aggregate level) and matrix reasoning scores in the ICAR (Condon & Revelle, 2014). The correlation was 0.54 [*t* (48) = 4.44, p < 0.001, 95% CI = 0.31 0.71], and acceptable for convergent validity purposes (Carlson & Herdman, 2010). Linear regression also showed that the correlation did not interact with the order in which participants completed the two tasks (p = 0.716). The performance standard deviations of the ICAR matrix reasoning items in our sample and in Condon & Revelle (2014) were 0.25 and 0.5, respectively, warranting a correction for age restriction (Condon & Revelle, 2014). The range corrected correlation was 0.79, thus again within acceptable range. To assess the extent of divergent validity, we further compared the correlation described above to the correlations between the MaRs-IB and the remaining tasks of the ICAR (Table S11), namely, letter-number series completion, verbal reasoning and 3D rotations. The highest correlation with MaRs-IB performance was indeed with the ICAR matrix reasoning (Table S10). However, comparing the correlations using the Fisher r-z transform revealed that the MaRs-IB-ICAR matrices correlation did not significantly differ from the others (all p_s_ > 0.24). Similar to our main analysis with N=100, this evidence suggests an acceptable degree of convergent validity between the MaRs-IB and ICAR-matrix reasoning, with correlation sizes that are similar to those observed between the ICAR and other cognitive ability tests, such as the Shipley 2 (Condon & Revelle, 2014). We observed no evidence of divergent validity from other IQ-related measures of the ICAR, suggesting that the MaRs-IB may tap into a broad cognitive functioning construct (however, the larger sample size revealed some evidence of divergent validity – see manuscript).

**Supplementary Figures**

**
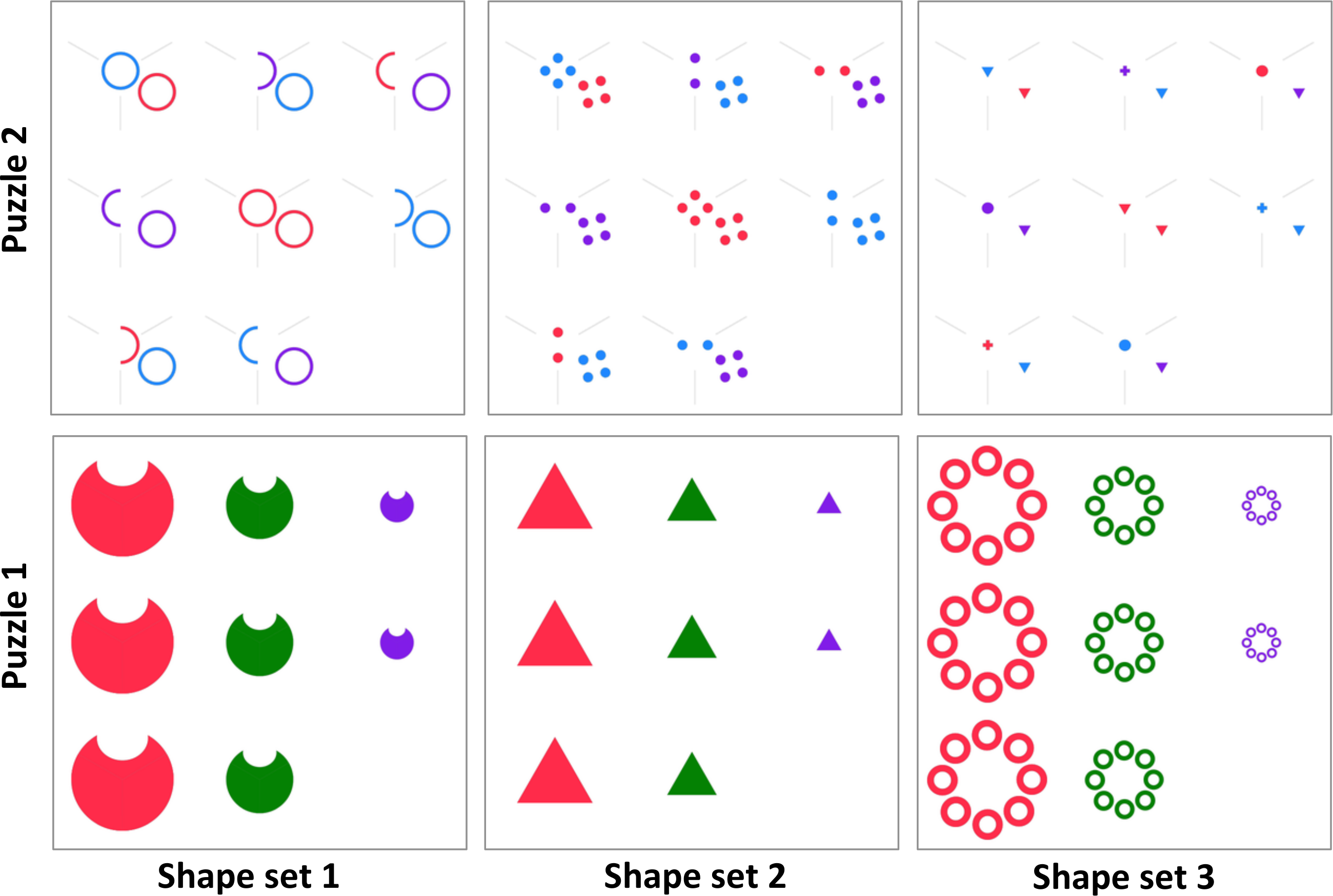
**

*Figure S1.* *Shape Sets*. Examples of the three different shape sets for two puzzles.


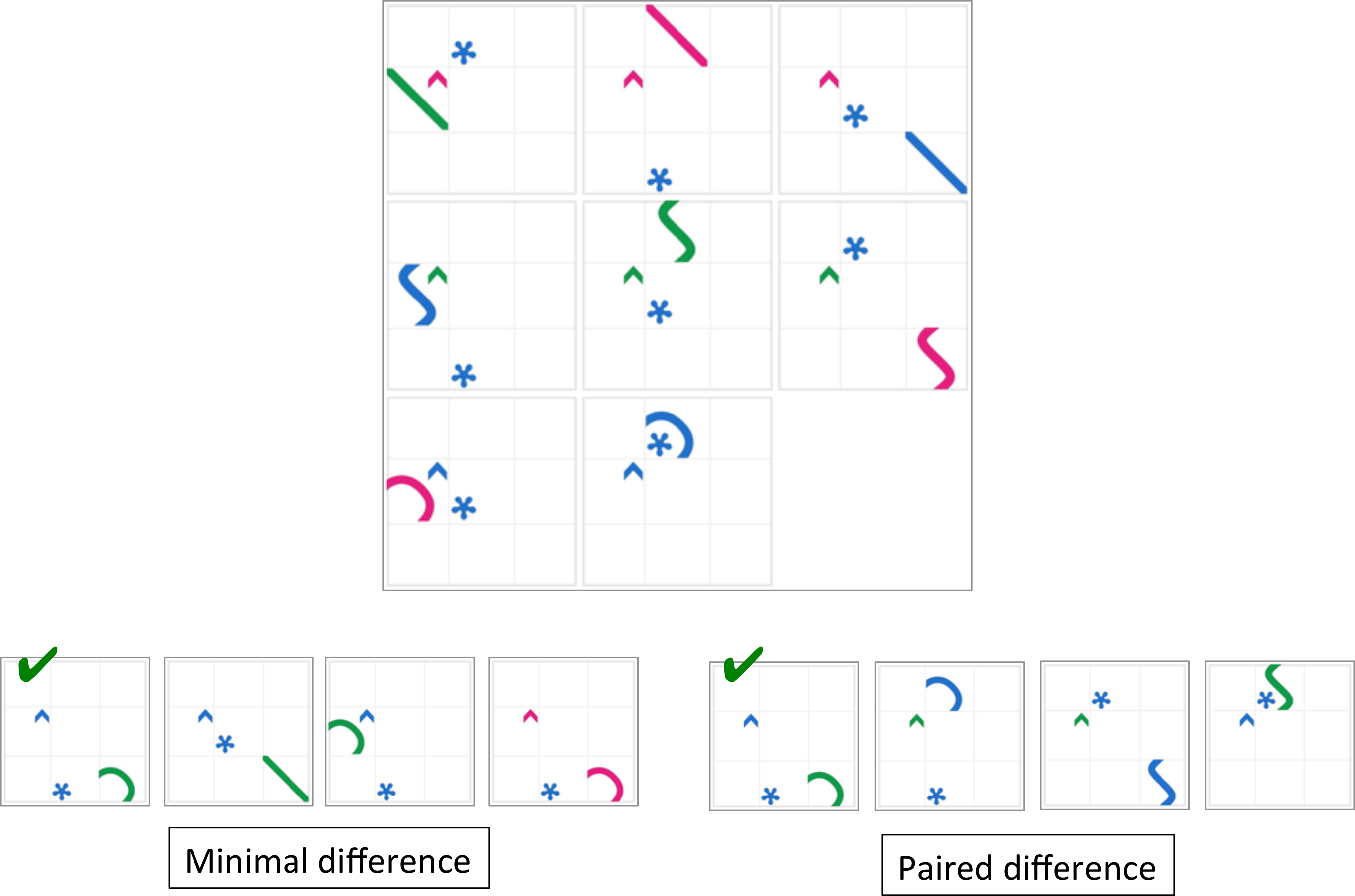


*Figure S2.* *Target and Distractor Generation.* The minimal difference strategy potentially allows solving the puzzle by looking at the solutions only (e.g., one of the solutions is “minimally different” from all the others). The paired difference strategy does not allow this but potentially allows “pop-out” effects. Green tick marks indicate the correct response option.

*
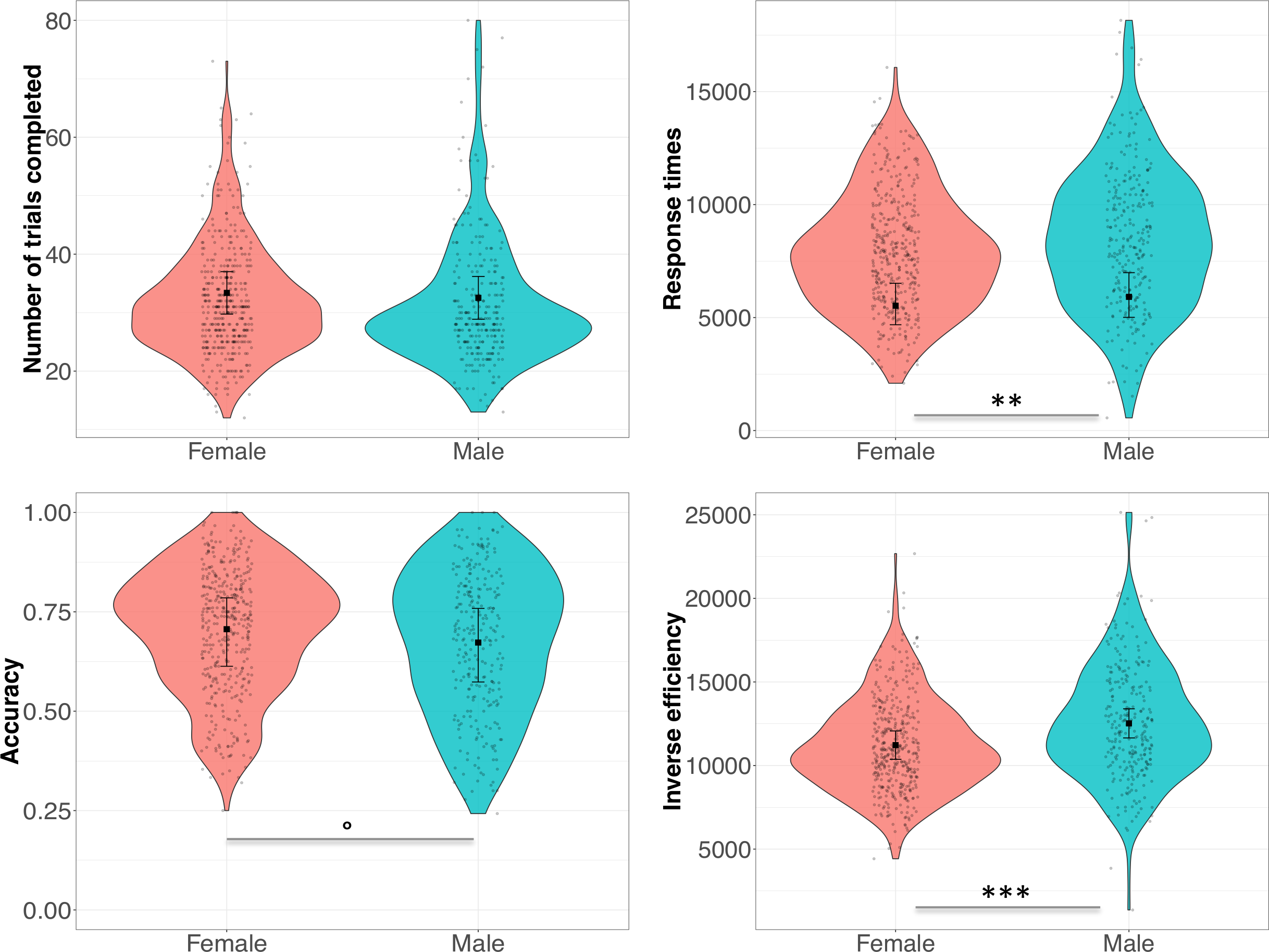
*

*Figure S3.* *Gender Differences in MaRs-IB Performance.* Figure shows the number of items completed, accuracy, response times and inverse efficiency. **°** *p* < 0.1, ** *p* < .01, *** *p* < .001.

**Supplementary Tables**

*Table S1. MaRs-IB Item Statistics.*

| **Item** | **N** | **Dim** | **P-value (SE)** | **RT (IQR)** | **IES** | **Item** | **N** | **Dim** | **P-value (SE)** | **RT (IQR)** | **IES** |
| --- | --- | --- | --- | --- | --- | --- | --- | --- | --- | --- | --- |
| 1 | 649 | 2 | 97 (1) | 2411 (1577) | 2486 | 41 | 113 | 1 | 84 (3) | 4248 (2618) | 5057 |
| 2 | 655 | 1 | 97 (1) | 4446 (2514) | 4584 | 42 | 106 | 4 | 27 (4) | 5674 (6414) | 21015 |
| 3 | 647 | 1 | 98 (1) | 2462 (1185) | 2512 | 43 | 91 | 1 | 33 (5) | 4720 (4510) | 14305 |
| 4 | 654 | 1 | 99 (0) | 3763 (1705) | 3801 | 44 | 83 | 6 | 19 (4) | 2424 (4126) | 12758 |
| 5 | 648 | 1 | 100 (0) | 2528 (1378) | 2528 | 45 | 73 | 7 | 33 (6) | 6316 (7412) | 19138 |
| 6 | 643 | 2 | 78 (2) | 9410 (7113) | 12064 | 46 | 61 | 6 | 38 (6) | 4185 (5728) | 11013 |
| 7 | 645 | 1 | 98 (1) | 5179 (2921) | 5285 | 47 | 57 | 2 | 21 (5) | 5035 (7364) | 23976 |
| 8 | 644 | 1 | 85 (1) | 7656 (5632) | 9006 | 48 | 49 | 1 | 33 (7) | 2942 (6569) | 8915 |
| 9 | 654 | 1 | 94 (1) | 5898 (3022) | 6274 | 49 | 47 | 2 | 47 (7) | 4181 (2674) | 8896 |
| 10 | 631 | 3 | 53 (2) | 15262 (10196) | 28795 | 50 | 46 | 2 | 37 (7) | 4021 (4195) | 10868 |
| 11 | 629 | 3 | 59 (2) | 13966 (9785) | 23670 | 51 | 41 | 2 | 29 (7) | 5712 (3462) | 19697 |
| 12 | 623 | 4 | 25 (2) | 16342 (12690) | 65366 | 52 | 35 | 5 | 37 (8) | 1757 (2072) | 4749 |
| 13 | 620 | 3 | 49 (2) | 17337 (10760) | 35382 | 53 | 30 | 4 | 20 (7) | 1286 (1058) | 6432 |
| 14 | 608 | 5 | 37 (2) | 18867 (12907) | 50992 | 54 | 30 | 6 | 13 (6) | 1612 (1382) | 12404 |
| 15 | 642 | 3 | 56 (2) | 13588 (11348) | 24264 | 55 | 28 | 6 | 36 (9) | 2770 (3133) | 7693 |
| 16 | 648 | 4 | 89 (1) | 7677 (6066) | 8626 | 56 | 25 | 3 | 20 (8) | 1726 (1443) | 8630 |
| 17 | 639 | 4 | 59 (2) | 13888 (10111) | 23539 | 57 | 21 | 1 | 43 (11) | 4151 (2261) | 9653 |
| 18 | 627 | 3 | 41 (2) | 15692 (11098) | 38273 | 58 | 21 | 3 | 43 (11) | 3836 (2070) | 8921 |
| 19 | 636 | 2 | 82 (2) | 7902 (5100) | 9637 | 59 | 20 | 5 | 25 (10) | 2806 (3091) | 11224 |
| 20 | 630 | 2 | 67 (2) | 12114 (7182) | 18081 | 60 | 14 | 4 | 50 (14) | 1944 (1010) | 3888 |
| 21 | 597 | 7 | 49 (2) | 16676 (9658) | 34033 | 61 | 16 | 2 | 12 (9) | 1590 (1312) | 13254 |
| 22 | 597 | 2 | 82 (2) | 7700 (4962) | 9390 | 62 | 15 | 3 | 27 (12) | 5922 (4046) | 21933 |
| 23 | 592 | 4 | 81 (2) | 9836 (6223) | 12143 | 63 | 14 | 5 | 14 (10) | 4792 (3122) | 34225 |
| 24 | 554 | 7 | 41 (2) | 15723 (11576) | 38349 | 64 | 11 | 6 | 36 (15) | 1010 (452) | 2807 |
| 25 | 543 | 2 | 77 (2) | 9332 (7017) | 12119 | 65 | 9 | 2 | 11 (11) | 849 (NA) | 7718 |
| 26 | 509 | 5 | 36 (2) | 17140 (10786) | 47611 | 66 | 9 | 8 | 22 (15) | 1194 (636) | 5430 |
| 27 | 478 | 3 | 54 (2) | 11507 (8290) | 21309 | 67 | 8 | 5 | 25 (16) | 768 (59) | 3072 |
| 28 | 439 | 2 | 57 (2) | 13078 (7606) | 22944 | 68 | 6 | 1 | 100 (0) | 1964 (527) | 1964 |
| 29 | 393 | 7 | 62 (2) | 11622 (8504) | 18745 | 69 | 7 | 3 | 57 (20) | 3776 (1369) | 6625 |
| 30 | 349 | 7 | 60 (3) | 9362 (5374) | 15603 | 70 | 8 | 2 | 50 (19) | 3495 (1420) | 6990 |
| 31 | 317 | 3 | 55 (3) | 11404 (7901) | 20735 | 71 | 6 | 2 | 50 (22) | 4439 (2121) | 8878 |
| 32 | 289 | 1 | 56 (3) | 8070 (6095) | 14412 | 72 | 5 | 4 | 40 (24) | 1164 (689) | 2910 |
| 33 | 254 | 1 | 60 (3) | 6414 (3698) | 10689 | 73 | 6 | 4 | 17 (17) | 1033 (NA) | 6076 |
| 34 | 234 | 3 | 37 (3) | 8640 (8548) | 23351 | 74 | 4 | 5 | 50 (29) | 4274 (3827) | 8548 |
| 35 | 211 | 8 | 42 (3) | 8321 (8830) | 19812 | 75 | 6 | 6 | 50 (22) | 989 (524) | 1978 |
| 36 | 192 | 6 | 36 (3) | 6823 (8381) | 18953 | 76 | 4 | 5 | 75 (25) | 507 (327) | 676 |
| 37 | 177 | 3 | 36 (4) | 8496 (9960) | 23600 | 77 | 3 | 3 | 33 (33) | 455 (NA) | 1379 |
| 38 | 156 | 1 | 83 (3) | 4240 (2509) | 5108 | 78 | 4 | 7 | 25 (25) | 631 (NA) | 2524 |
| 39 | 135 | 2 | 41 (4) | 5448 (7342) | 13288 | 79 | 5 | 4 | 0 (0) | NA | NA |
| 40 | 122 | 4 | 44 (5) | 5020 (4839) | 11409 | 80 | 3 | 4 | 33 (33) | 640 (NA) | 1939 |

*Note.* N = number of responses; Dim = dimensionality; RT = median response time of correct responses (with interquartile range); IES = inverse efficiency;

NA = not applicable (too few observations)

*Table S2. MaRs-IB Item Statistics on Items with Invariant completion Rate.*

| **Item** | **N** | **Dim** | **P-value (SE)** | **RT (IQR)** | **IES** | **ITC** |
| --- | --- | --- | --- | --- | --- | --- |
| 6 | 349 | 2 | 75 (2) | 8068 (5326) | 10757 | 0.3 |
| 7 | 349 | 1 | 97 (1) | 4679 (2224) | 4824 | 0.13 |
| 8 | 349 | 1 | 81 (2) | 6530 (5011) | 8062 | 0.4 |
| 9 | 349 | 1 | 90 (2) | 5506 (2811) | 6118 | 0.31 |
| 10 | 349 | 3 | 50 (3) | 13642 (7592) | 27284 | 0.4 |
| 11 | 349 | 3 | 56 (3) | 11357 (7355) | 20280 | 0.25 |
| 12 | 349 | 4 | 19 (2) | 12684 (7888) | 66758 | 0.26 |
| 13 | 349 | 3 | 44 (3) | 14655 (9240) | 33307 | 0.23 |
| 14 | 349 | 5 | 33 (3) | 14436 (10613) | 43745 | 0.28 |
| 15 | 349 | 3 | 50 (3) | 11210 (7235) | 22420 | 0.31 |
| 16 | 349 | 4 | 86 (2) | 6521 (4230) | 7583 | 0.31 |
| 17 | 349 | 4 | 55 (3) | 11705 (8786) | 21282 | 0.19 |
| 18 | 349 | 3 | 31 (2) | 12776 (8538) | 41213 | 0.29 |
| 19 | 349 | 2 | 78 (2) | 6914 (4294) | 8865 | 0.35 |
| 20 | 349 | 2 | 56 (3) | 9958 (6473) | 17781 | 0.48 |
| 21 | 349 | 7 | 44 (3) | 13687 (8355) | 31107 | 0.42 |
| 22 | 349 | 2 | 79 (2) | 6585 (4076) | 8335 | 0.33 |
| 23 | 349 | 4 | 77 (2) | 8713 (4846) | 11316 | 0.4 |
| 24 | 349 | 7 | 34 (3) | 12947 (10951) | 38079 | 0.21 |
| 25 | 349 | 2 | 72 (2) | 8342 (4811) | 11587 | 0.36 |
| 26 | 349 | 5 | 34 (3) | 14413 (11665) | 42391 | 0.18 |
| 27 | 349 | 3 | 51 (3) | 10591 (6562) | 20767 | 0.38 |
| 28 | 349 | 2 | 55 (3) | 12354 (7528) | 22462 | 0.33 |
| 29 | 349 | 7 | 60 (3) | 10856 (8430) | 18094 | 0.4 |
| 30 | 349 | 7 | 60 (3) | 9362 (5374) | 15603 | 0.43 |

Note. N = number of responses; Dim = dimensionality; RT = median response time of correct responses (with interquartile range); IES = inverse efficiency; ITC = item-total correlation. None of the items displayed uniform or non-uniform differential item functioning relative to age and gender.

*Table S3. Descriptive Statistics of the MaRs-IB by Test Form.*

|  | **puzzle set 1** | **puzzle set 2** | **puzzle set 3** |
| --- | --- | --- | --- |
| RT (median) | 8372 | 7530 | 7790 |
| RT (IQR) | 3606.0 | 4052 | 4339 |
| Accuracy (mean) | 69 | 69 | 69 |
| Accuracy (SE) | 1 | 1 | 1 |
| Accuracy (Min) | 27 | 25 | 24 |
| Accuracy (Max) | 100 | 100 | 100 |
| Items completed (mean) | 31.29 | 32.32 | 32.19 |
| Items completed (SE) | 0.70 | 0.61 | 0.74 |
| Items completed (Min) | 13 | 12 | 14 |
| Items completed (Max) | 77 | 64 | 80 |

*Table S4. Descriptive Statistics of the MaRs-IB by Shape Set.*

|  | **shape set 1** | | **shape set 2** | **shape set 3** |
| --- | --- | --- | --- | --- |
| RT (median) | 7714 | | 8199 | 7855 |
| RT (IQR) | 4549 | | 5235 | 5325 |
| Accuracy (mean) | | 70 | 69 | 68 |
| Accuracy (SE) | | 1 | 1 | 1 |
| Accuracy (Min) | | 10 | 9 | 18 |
| Accuracy (Max) | | 100 | 100 | 100 |
| Items completed (mean) | | 10.65 | 10.66 | 10.63 |
| Items completed (SE) | | 0.13 | 0.13 | 0.14 |
| Items completed (Min) | | 4 | 4 | 3 |
| Items completed (Max) | | 27 | 27 | 26 |

*Table S5. Descriptive Statistics of the MaRs-IB by Distractor Strategy.*

|  | **minimal difference** | **paired difference** |
| --- | --- | --- |
| RT (median) | 7853 | 7969 |
| RT (IQR) | 4344 | 4781 |
| Accuracy (mean) | 69 | 69 |
| Accuracy (SE) | 1 | 1 |
| Accuracy (Min) | 19 | 18 |
| Accuracy (Max) | 100 | 100 |
| Items completed (mean) | 15.96 | 15.98 |
| Items completed (SE) | 0.2 | 0.2 |
| Items completed (Min) | 5 | 5 |
| Items completed (Max) | 40 | 40 |

*Table S6. Accuracy: Age Group Contrasts.*

| ***contrast*** | ***estimate*** | ***SE*** | ***p*_Bonf._** | ***p*_uncorr._** |
| --- | --- | --- | --- | --- |
| Younger adolescents - Mid adolescents | -0.28 | 0.13 | .163 | .027 |
| Younger adolescents - Older adolescents | -0.33 | 0.2 | .591 | .099 |
| Younger adolescents - Adults | -1.13 | 0.4 | .027 | .004 |
| Mid adolescents - Older adolescents | -0.06 | 0.16 | 1 | .728 |
| Mid adolescents - Adults | -0.85 | 0.38 | .158 | .026 |
| Older adolescents - Adults | -0.8 | 0.38 | .219 | .036 |

*Note.* Results are shown on the log odds scale.

*Table S7. Response Times: Age group Contrasts.*

| ***contrast*** | ***estimate*** | ***SE*** | ***p*_Bonf._** | ***p*_uncorr._** |
| --- | --- | --- | --- | --- |
| Younger adolescents - Mid adolescents | 0.03 | 0.04 | 1 | .542 |
| Younger adolescents - Older adolescents | 0.01 | 0.06 | 1 | .872 |
| Younger adolescents - Adults | -0.11 | 0.12 | 1 | .357 |
| Mid adolescents - Older adolescents | -0.01 | 0.05 | 1 | .783 |
| Mid adolescents - Adults | -0.14 | 0.12 | 1 | .253 |
| Older adolescents - Adults | -0.12 | 0.12 | 1 | .317 |

*Note.* Results are shown on the log scale.

*Table S8. Number of Items Completed: Age Group Contrasts.*

| ***contrast*** | ***estimate*** | ***SE*** | ***p*_Bonf._** | ***p*_uncorr._** |
| --- | --- | --- | --- | --- |
| Younger adolescents - Mid adolescents | 0.93 | 1.31 | 1 | .478 |
| Younger adolescents - Older adolescents | 0.12 | 2.02 | 1 | .951 |
| Younger adolescents - Adults | 1.51 | 5.17 | 1 | .772 |
| Mid adolescents - Older adolescents | -0.81 | 1.66 | 1 | .625 |
| Mid adolescents - Adults | 0.58 | 5.11 | 1 | .91 |
| Older adolescents - Adults | 1.39 | 5.11 | 1 | .788 |

*Table S9. Inverse Efficiency: Age Group Contrasts.*

| ***contrast*** | ***estimate*** | ***SE*** | ***p*_Bonf._** | ***p*_uncorr._** |
| --- | --- | --- | --- | --- |
| Younger adolescents - Mid adolescents | 762.94 | 413.23 | .395 | .066 |
| Younger adolescents - Older adolescents | 844.14 | 612.87 | 1 | .171 |
| Younger adolescents - Adults | 681.36 | 1317.95 | 1 | .612 |
| Mid adolescents - Older adolescents | 81.2 | 515.11 | 1 | .875 |
| Mid adolescents - Adults | -81.57 | 1300.44 | 1 | .951 |
| Older adolescents - Adults | -162.77 | 1318.15 | 1 | .903 |

*Table S10. Correlations between MaRs-IB and tasks from the International Cognitive Ability Resource*

|  | **MaRs-IB** | **MR** | **R3D** | **LN** |
| --- | --- | --- | --- | --- |
| **MaRs-IB** | - |  |  |  |
| **MR** | 0.54*** | - |  |  |
| **R3D** | 0.43* | 0.29 | - |  |
| **LN** | 0.39° | 0.46** | 0.35 | - |
| **VR** | 0.45** | 0.52** | 0.38° | 0.37° |

*Note.* MR = matrix reasoning; R3D = 3D rotations; LN = letter and number series completion; VR = verbal reasoning. *** *p* < 0.001, ** *p* < 0.01, * *p* < 0.05, ° *p*<0.1. Bonferroni corrected.
